# Genome-Wide Identification of CRK Family Members in Solanaceae Crops and Functional Characterization of *CaCRK10* and *SmCRK6* in Salt Stress Response

**DOI:** 10.3390/cimb48090965

**Published:** 2026-09-21

**Authors:** Jingyi Lyu, Dongrun Yu, Yiru Zhang, Qi Chen, Wenhao Hu, Yu Xin, Fengjuan Yang, Guobin Yang, Tuo Ji

**Affiliations:** 1 Future Technology College (Qilu Academy), Shandong Agricultural University, Tai’an 271018, China; jyinglv826@163.com (J.L.); dryu13053902563@163.com (D.Y.); 2 College of Horticulture Science and Engineering, Shandong Agricultural University, Tai’an 271018, China; 17863855817@163.com (Y.Z.); chihuoxiaochen@163.com (Q.C.); hu20000930@outlook.com (W.H.); 15863554836@163.com (Y.X.); fjyang@sdau.edu.cn (F.Y.); 3 Key Laboratory of Biology and Genetic Improvement of Horticultural Crops in Huang-Huai Region, Ministry of Agriculture and Rural Affairs, Tai’an 271018, China; 4 Shandong Key Laboratory of Fruit and Vegetable Germplasm Innovation and Utilization, Tai’an 271018, China; 5 College of Mechanical Engineering, Taishan University, Tai’an 271000, China; 6 National Center of Technology Innovation for Comprehensive Utilization of Saline-Alkali Land, Agricultural High-Tech Industrial Demonstration Area of the Yellow River Delta of Shandong Province, Dongying 257000, China

**Keywords:** cysteine-rich receptor-like kinase (CRK), Solanaceae crops, salt stress tolerance, ion homeostasis, phosphorylation

## Abstract

Salt stress severely limits crop growth and productivity, particularly in horticultural crops. Cysteine-rich receptor-like kinases (CRKs) are important membrane-associated signaling proteins involved in plant environmental responses; however, their functions in salt stress adaptation in Solanaceae crops remain limited. In this study, a genome-wide identification and comparative analysis of the *CRK* gene family was performed in pepper (*Capsicum annuum*), eggplant (*Solanum melongena*), and tomato (*Solanum lycopersicum*). 37 CRK genes were identified in pepper. Comprehensive analyses, including phylogenetic relationships, conserved domain organization, motif composition, and evolutionary patterns, demonstrated that CRK proteins in Solanaceae share highly conserved structural characteristics, consisting of extracellular DUF26 domains, transmembrane regions, and intracellular serine/threonine kinase domains. Expression analysis revealed that multiple CRK genes responded to salt stress, among which *CaCRK10* showed strong induction and was selected for functional characterization. Subcellular localization analysis indicated that CaCRK10 was mainly localized to the plasma membrane. Overexpression of *CaCRK10* improved the salt tolerance of pepper plants, as indicated by stronger antioxidant activity and lower malondialdehyde accumulation under salt stress. The transgenic plants also showed a better balance between K^+^ and Na^+^, together with improved osmotic adjustment. In addition, *CaCRK10* overexpression increased the transcript levels of several SOS- and NHX-related genes associated with ion homeostasis. A similar response was observed for the eggplant homolog *SmCRK6*, whose expression was strongly induced by salt treatment. Its overexpression also contributed to improved salt tolerance, which was associated with changes in antioxidant defense, ion homeostasis, and osmotic regulation. Together, these findings initially revealed that CRK members play conserved positive regulatory roles in salt stress adaptation in Solanaceae crops and provide potential genetic resources for molecular breeding of salt-tolerant varieties.

## 1. Introduction

Solanaceae crops are among the most important horticultural crops worldwide, especially pepper, eggplant, tomato and potato, which possess significant economic and agricultural value. These crops are cultivated extensively in many parts of the world and make important contributions to agricultural production, as they are widely consumed as part of the human diet and generate considerable economic value [1,2]. However, the development of crops is frequently restricted by various abiotic stresses [3], including salinity, drought, low temperature, and other environmental constraints. Among these stresses, salt stress is one of the major environmental factors limiting global agricultural production and sustainable crop development [4,5]. Excessive salt accumulation induces osmotic stress in the rhizosphere; it will disrupt ion homeostasis and promote excessive accumulation of reactive oxygen species (ROS), causing cellular membrane damage, ultimately impairing photosynthesis and yield formation [4,6].

In recent years, the cultivation area of Solanaceae crops under non-protected field conditions in China has continuously expanded [7]. Meanwhile, saline–alkali land has attracted increasing attention as a potential agricultural resource. China possesses approximately 1.5 billion mu of saline–alkali land, accounting for about 10% of the global saline–alkali land area, which is widely distributed across the central and western regions, northeastern areas, and eastern coastal regions. Among these areas, approximately 500 million mu of saline–alkali land has potential for agricultural development and utilization, and some mildly saline–alkaline soils can even be directly used for crop cultivation [8]. Interestingly, some horticultural crops exhibit improved quality under moderate saline–alkaline conditions. Previous studies have shown that salt stress significantly increases the soluble solids content of tomato fruits [9]. Xu et al. reported that abscisic acid (ABA) produced in tomato roots under salt stress promotes sucrose metabolism and sugar accumulation, enhancing tomato fruit sweetness [10]. Moreover, tomatoes cultivated under saline–alkali conditions exhibit higher accumulation of quality-related compounds, including vitamin C, carotenoids, soluble sugars, organic acids, and volatile aromatic substances, compared with conventionally cultivated tomatoes [9,11,12]. Therefore, the identification and breeding of high-quality Solanaceae crop varieties adapted to saline–alkali environments, while balancing yield and quality requirements, are of great significance for improving the utilization efficiency of non-arable land and ensuring stable horticultural crop production [6].

Genome-wide identification and characterization of stress-related gene families have become important approaches for dissecting the genetic basis of plant adaptation to abiotic stresses and for identifying candidate genes with potential value in crop improvement. By integrating phylogenetic relationships, gene structure, conserved domains, chromosomal distribution, and stress-responsive expression profiles, genome-wide studies can provide a systematic view of gene family expansion and functional diversification and help prioritize candidate genes for subsequent functional validation. In crops, genome-wide analyses of transcription factors and stress-related gene families have identified candidate regulators associated with salt, drought, heat, and other abiotic stresses, providing genetic resources for stress-resilient breeding [13]. For example, genome-wide analyses of heat shock factor families in eggplant revealed extensive structural diversity and differential responses to multiple abiotic stresses [14], while genome-wide characterization of the DREB family in pepper identified numerous genes responsive to salt and osmotic stresses and further identified *CaDREB32* as a candidate regulator of stress tolerance [15]. These studies demonstrate that genome-wide characterization can serve as an effective framework for linking genome-scale gene diversity with stress-responsive phenotypes and for selecting candidate genes for functional and breeding applications.

During long-term evolution, plants have developed complex signal perception and transduction networks to respond to environmental changes and activate self-defense mechanisms [16]. Receptor-like kinases (RLKs) are important membrane proteins that perceive external environmental signals and initiate downstream signal transduction, playing crucial roles in plant growth and development like immune responses and abiotic stress adaptation [17]. Among them, cysteine-rich receptor-like kinases (CRKs) constitute a large subgroup of DUF26-containing receptor-like kinase (RLK) families. CRKs typically consist of an extracellular domain, a transmembrane domain (TMD), and an intracellular serine/threonine kinase domain. The extracellular region contains one to four DUF26 motifs, whose core structure is characterized by a conserved cysteine-rich motif (C-X8-C-X2-C) [18]. CRKs have been reported to participate in diverse biological processes, including plant immunity and reactive oxygen species signaling. Through ligand perception and receptor dimerization, RLKs activate downstream signaling modules involved in gene expression regulation, stomatal aperture control, and hormone signaling pathways. For example, the transcript abundance of *CRK1* in *Triticum aestivum* was increased following Fusarium infection and exogenous ABA treatment. OsRMC, a protein containing extracellular DUF26 domains, has been reported to participate in root bending and salt stress responses in *Oryza sativa* L. [19]. In *Arabidopsis thaliana*, CRK45 functions as a positive regulator of disease resistance, as CRK45 overexpression enhances resistance to *Pseudomonas syringae*, whereas crk45 mutants exhibit increased susceptibility to *P. syringae* infection [20]. SCR96, a cysteine-rich small protein derived from *Phytophthora cactorum*, plays important roles in pathogenicity and oxidative stress responses [21]. The functional diversity of CRKs is largely attributed to variations in extracellular recognition domains and precise regulation of kinase activity [22]. However, systematic studies on the roles of CRK family members in salt stress responses of Solanaceae crops remain scarce, and the molecular mechanisms underlying CRK-mediated salt tolerance have not yet been fully elucidated. Therefore, systematic identification of CRK family members in Solanaceae crops is essential for understanding the molecular mechanisms of salt tolerance. By characterizing their salt stress-responsive patterns and exploring key regulatory genes, such studies may provide valuable insights into the development of salt-tolerant crop varieties.

Previous studies have systematically analyzed the CRK gene family in potato and revealed that *StCRLK9* responds to multiple abiotic stresses, including heat, salt, and drought stress, providing preliminary insights into its biological function [23]. However, comprehensive studies of the CRK gene family in other economically important Solanaceae crops remain limited. Pepper and eggplant were selected as the primary horticultural crops for functional investigation because both are economically important Solanaceae vegetables and are frequently exposed to environmental stresses that can constrain growth and productivity. In addition, their available genome resources provide a suitable basis for systematic gene-family identification and comparative evolutionary analysis. Previous genome-wide studies in pepper and eggplant have successfully identified stress-responsive members of several gene families, demonstrating the feasibility of using these two crops for genome-scale exploration of abiotic stress regulators. However, compared with transcription factors and other well-characterized stress-related gene families, the CRK family remains insufficiently investigated in these two horticultural crops, particularly with respect to salt stress. Therefore, pepper and eggplant provide complementary systems for examining whether CRK-mediated salt stress responses are conserved across distinct Solanaceae crops while also allowing species-specific candidate genes to be identified. This study performed a systematic identification and comparative analysis of the CRK gene family in representative Solanaceae crops and preliminarily characterized the functions of salt-responsive CRK genes in two horticultural crops, pepper (*Capsicum annuum*) and eggplant (*Solanum melongena*). Through genome-wide identification, phylogenetic analysis, conserved domain analysis, and expression pattern analysis, this study revealed the composition characteristics, evolutionary relationships, and salt stress-responsive patterns of CRK family members in Solanaceae crops, particularly pepper and eggplant. Transcriptome data and expression analyses indicated that most CRK genes are regulated by various environmental stresses, suggesting that this family may broadly participate in stress-responsive signaling pathways in Solanaceae plants. Furthermore, functional analysis identified the pepper candidate gene *CaCRK10* and demonstrated its important role in salt stress responses. Overexpression of *CaCRK10* significantly enhanced salt tolerance in pepper plants, mainly through increasing antioxidant enzyme activities, promoting the expression of antioxidant-related genes, and maintaining reactive oxygen species (ROS) homeostasis, thereby reducing salt-induced oxidative damage and improving plant adaptation to saline environments [4]. In addition, this study further investigated the eggplant CRK member *SmCRK6* and found that it also participates in salt stress responses, suggesting that CRK members from different Solanaceae crops may share certain functional associations and jointly contribute to plant adaptation to adverse environments. This study expands our understanding of receptor-like kinase-mediated salt stress signaling mechanisms in Solanaceae crops and provides potential genetic resources for breeding salt-tolerant pepper and eggplant varieties.

## 2. Materials and Methods

### 2.1. Identification and Chromosomal Distribution Analysis of the CaCRK Gene Family in Pepper

The pepper genome sequence (Zunla-1_v3.0.genome.fasta) was downloaded from PepperBase (http://www.bioinformaticslab.cn/PepperBase/download/, accessed on 18 May 2026), protein sequence database (Zunla-1_v3.0.genome.pep) was obtained from PepperBase (http://www.bioinformaticslab.cn/PepperBase/download/, accessed on 18 May 2026), and the corresponding genome annotation file (Zunla-1_v3.0.genome.gff3) was obtained from PepperBase (http://www.bioinformaticslab.cn/PepperBase/download/, accessed on 19 May 2026). Hidden Markov Model (HMM) profiles of the kinase domain (PF00069) and stress-responsive antifungal domain (PF01657) were downloaded from the Pfam database (https://pfam.xfam.org/, accessed on 20 May 2026). The preliminary identification of candidate CRK genes in the *Capsicum annuum* genome was performed using BLASTP (Basic Local Alignment Search Tool for proteins) based on CRK protein sequences retrieved from The Arabidopsis Information Resource (TAIR, https://www.arabidopsis.org/, accessed on 20 May 2026), with an E-value threshold of 0.001. The candidate sequences obtained from BLASTP analysis were further screened using PfamScan (E-value = 0.001; https://www.ebi.ac.uk/Tools/pfa/pfamscan/, accessed on 20 May 2026) and the NCBI Conserved Domain Database (NCBI-CDD, E-value = 0.001; https://www.ncbi.nlm.nih.gov/cdd/, accessed on 21 May 2026). Redundant sequences and sequences lacking conserved CRK domains were removed. Basic information of *CaCRK* genes, including chromosomal localization, number of introns, average intron length, protein length, and isoelectric point (pI), was obtained from the genome annotation database. Chromosomal distribution of *CaCRK* genes was visualized using TBtools (South China Agricultural University, Guangzhou, China; https://github.com/CJ-Chen/Tbtools, accessed on 21 May 2026). Similar identification procedures were applied to eggplant (*S. melongena*) and tomato (*S. lycopersicum*) using their respective genome databases.

### 2.2. Phylogenetic Analysis of the CRK Gene Family

The amino acid sequences of CRK family members from *S. lycopersicum*, *S. melongena*, *C. annuum* and *A. thaliana* were aligned using Clustal X (University College Dublin, Dublin, Ireland). A neighbor-joining (NJ) phylogenetic tree was constructed using MEGA 12 (Molecular Evolutionary Genetics Analysis, Tempe, AZ, USA; www.megasoftware.net, accessed on 21 May 2026) with 1000 bootstrap replicates.

### 2.3. Gene Structure, Conserved Motif Identification, and Collinearity Analysis

The exon–intron structures of *CaCRK* genes were analyzed using the Gene Structure Display Server (GSDS, http://gsds.gao-lab.org/, accessed on 22 May 2026). Conserved motifs in CaCRK proteins were identified using MEME Suite version 5.5.8 (University of Nevada, Reno, NV, USA; http://meme-suite.org/, accessed on 22 May 2026). The collinearity relationships of CRK genes among *C. annuum*, *S. melongena*, *A. thaliana* [24], and *S. lycopersicum* were analyzed using MCScanX (University of Georgia, Athens, GA, USA; https://github.com/wyp1125/MCScanX, accessed on 22 May 2026). The results were visualized using TBtools (https://github.com/CJ-Chen/Tbtools, accessed on 23 May 2026).

### 2.4. Prediction of Protein Tertiary Structures

The tertiary structures of pepper CRK proteins were predicted using the AlphaFold Protein Structure Database (DeepMind, London, UK; https://alphafold.com/, accessed on 25 May 2026). The reliability of AlphaFold-predicted models was evaluated based on the predicted Local Distance Difference Test (pLDDT) score for each amino acid residue. A pLDDT score above 90 indicates a very high-confidence prediction, scores between 70 and 90 indicate high confidence, scores between 50 and 70 indicate low confidence, and scores below 50 represent regions with poor prediction reliability. The overall model quality was assessed using the predicted template modeling (pTM) score. The predicted positional errors between residues were evaluated using predicted aligned error (PAE) heatmaps.

### 2.5. Expression Analysis of the CRK Family Under Salt Stress

Public RNA-seq datasets PRJNA649852 and PRJNA296071 (https://www.ncbi.nlm.nih.gov/bioproject, accessed on 25 May 2026) were retrieved from the NCBI Sequence Read Archive (SRA) to analyze the expression patterns of CRK family genes in *Arabidopsis thaliana* and *Oryza sativa* under salt stress conditions. Fragments per kilobase of transcript per million mapped reads (FPKM) values were extracted and used to evaluate transcript abundance. Expression heatmaps were generated using TBtools version 2.0 to visualize differential expression patterns among different samples and treatments.

### 2.6. Plant Materials and Treatments

Pepper and eggplant seedlings were cultivated under controlled growth conditions (28 °C/18 °C). For salt stress treatment, one-month-old seedlings with uniform growth were selected and treated with 100 mM NaCl dissolved in 1/2 Hoagland nutrient solution. This concentration was selected because 100 mM NaCl has been widely used to induce a reproducible and physiologically relevant salt stress response in pepper and eggplant without causing excessive tissue damage during short- to medium-term treatments. Previous studies have shown that 100 mM NaCl can induce clear changes in growth, ion homeostasis, osmotic adjustment, and oxidative stress-related physiological responses in these Solanaceae crops [25]. Therefore, this concentration was used in the present study to provide sufficient salt stress for evaluating transcriptional and physiological responses while maintaining plant viability throughout the treatment period. Samples were collected at 0, 6, and 12 h, as well as 1, 3, and 6 days after treatment. All samples were rapidly frozen in liquid nitrogen and stored at −80 °C until RNA extraction. Three biological replicates were performed for each treatment and tissue type, with each replicate representing an independent plant.

### 2.7. Quantitative Real-Time PCR Analysis

Total RNA was extracted from the roots of pepper and eggplant seedlings using an RNA extraction kit (Vazyme, Beijing, China). Quantitative real-time PCR (qRT-PCR) analysis was performed using an ABI PRISM 7500 Real-Time PCR System (Applied Biosystems, Foster City, CA, USA) according to the previously described 2^−ΔΔCT^ method [26]. Specific primers for *CaCRK* genes used in this study are listed in Table S1. The expression levels of target genes were normalized using two internal reference genes, actin and tubulin, in quantitative real-time PCR experiments. Specifically, *CaActin* (*ZLC12G0018530*) and *CaTubulin* (*ZLC04G0001910*) were used for pepper, while *SmActin* (*SMEL4.1_10g011330*) and *SmTubulin* (*SMEL4.1_10g011220*) were used for eggplant.

### 2.8. Subcellular Localization Analysis

The *CaCRK10* gene was cloned into the pCAMBIA1300 vector. The empty vector pCAMBIA1300-GFP were used as negative controls. The pCAMBIA1300 control and pCAMBIA1300-*CaCRK10* constructs were transformed into *Agrobacterium tumefaciens* strain GV3101, and the transformed bacterial suspensions were infiltrated into tobacco epidermal cells as previously described [27]. The localization of GFP fluorescence signals was observed using confocal laser scanning microscopy. Subcellular localization images were captured using a Zeiss LSM880 laser scanning confocal microscope (Zeiss, Jena, Germany).

### 2.9. Generation of Agrobacterium Rhizogenes-Mediated Composite Plants Through Hairy Root Transformation

To functionally evaluate whether *CaCRK10* and *SmCRK6* contribute to salt stress tolerance in pepper and eggplant, respectively, Agrobacterium rhizogenes-mediated hairy root transformation was used to generate composite plants overexpressing the candidate genes. This approach enabled rapid functional assessment of the selected CRK genes in transformed roots and was therefore used as a complementary strategy to the genome-wide identification and salt-responsive expression analyses. Four-week-old pepper and eggplant seedlings with uniform growth were selected for hairy root transformation. The primary roots were removed under sterile conditions, and the wounded stem bases were immersed in an *Agrobacterium rhizogenes* K599 suspension for 15 min [28]. Seedlings infected with K599 carrying the empty vector were used as negative controls. After inoculation, plants were transferred into moist vermiculite, covered to maintain high humidity, and co-cultivated in darkness at 28 °C for 3 days. Subsequently, plants were transferred to a growth chamber (28 °C, 16 h light/8 h dark photoperiod) and irrigated daily with 50 mL Hoagland nutrient solution to promote hairy root development.

### 2.10. Determination of Phenotypic and Physiological Traits Under Salt Stress

Uniform four-leaf-stage pepper and eggplant seedlings overexpressing *CaCRK10* or *SmCRK6* (OE) and vector control (VC) seedlings were cultured hydroponically and acclimated for 2 days. Subsequently, seedlings were treated with 100 mM NaCl or maintained under control conditions. For phenotypic evaluation, 14 biological replicates were used (*n* = 14). For physiological measurements, three biological replicates were analyzed (*n* = 3), with each biological replicate containing at least three independent seedlings.

Several physiological parameters were measured in this study. Malondialdehyde (MDA) content was determined using the thiobarbituric acid (TBA) colorimetric method [29] and expressed as nmol/g FW. Antioxidant enzyme activities were determined using corresponding methods. Superoxide dismutase (SOD) activity was measured using the nitro blue tetrazolium (NBT) photoreduction method and expressed as U/g FW. Peroxidase (POD) activity was determined using the guaiacol method and expressed as U/g FW [30]. After sample digestion, Na^+^ and K^+^ contents were measured using a flame photometer and expressed as μmol/g FW. Proline content was quantified using the acid ninhydrin colorimetric method [31] and expressed as μg/g FW. The determination of soluble sugars was carried out using the soluble sugar content detection kit (G0501F, GRACE Biotechnology, Suzhou, China), and was expressed as mg/g FW.

### 2.11. Analysis of Conserved Protein Structures and Prediction of Post-Translational Modification Sites in SmCRK6 and CaCRK10

To further investigate the structural conservation and potential functional association between SmCRK6 and CaCRK10 proteins, conserved motif prediction was performed using the MEME Suite version 5.5.8 (University of Nevada, Reno, NV, USA; http://meme-suite.org/, accessed on 22 May 2026). Based on the amino acid sequence characteristics of the two proteins, appropriate parameters were set to identify potential conserved motifs. The number, type, and arrangement order of conserved motifs in SmCRK6 and CaCRK10 proteins were subsequently compared. The structural conservation between SmCRK6 and CaCRK10 was evaluated by analyzing the distribution patterns of conserved motifs within the two proteins. Furthermore, sequence similarity analysis was performed to compare the conservation degree of different regions between SmCRK6 and CaCRK10 proteins. A sequence similarity heatmap was generated to identify highly conserved regions and potential functionally related domains within the two protein sequences. To predict potential post-translational modification sites in SmCRK6 and CaCRK10 proteins, the NetPhos 3.1 online prediction tool was used to identify potential phosphorylation sites of serine (Ser), threonine (Thr), and tyrosine (Tyr) residues within the protein sequences [32]. Based on the prediction results, the number and distribution positions of potential phosphorylation sites in the two proteins were statistically analyzed. The similarity of phosphorylation modification patterns between SmCRK6 and CaCRK10 was further compared to infer potential conserved regulatory mechanisms shared by these two proteins.

### 2.12. Statistical Analysis

All statistical analyses were performed using DPS version 22.05 software [33]. Graphs were generated using GraphPad Prism version 10.1.2 (GraphPad Software, San Diego, CA, USA). For comparisons between two groups, a two-tailed Student’s *t*-test was used. All data are presented as mean ± standard deviation (SD) from three independent biological replicates, with each replicate derived from a single plant or independent transformation event (*n* = 3). Statistical significance was defined as * *p* < 0.05, ** *p* < 0.01, *** *p* < 0.001, and **** *p* < 0.0001.

## 3. Results

### 3.1. Identification and Phylogenetic Analysis of CRKs in Solanaceae Crops

To obtain comprehensive CRK sequences from Solanaceae crops, genome-wide searches were performed against the protein databases of *Capsicum annuum* (pepper), *Solanum lycopersicum* (tomato), and *Solanum melongena* (eggplant) using HMMER based on the conserved domains of DUF26 (Pfam domain PF01657) and protein kinase domains (Pfam domains PF00069 and PF01657). The identified candidate proteins were further verified through Pfam domain analysis. CRK proteins possess typical receptor-like kinase structural characteristics, including two extracellular DUF26 domains, a transmembrane domain, and an intracellular serine/threonine protein kinase domain. Therefore, candidate proteins containing complete DUF26 domains and protein kinase domains were identified as CRK family members and designated as CaCRKs, SlCRKs, and SmCRKs in *C. annuum*, *S. lycopersicum*, and *S. melongena*, respectively. Finally, a total of 37 CRK family members were identified in the pepper genome (*C. annuum*) (Figure S1, Table S2). The encoded proteins ranged from 179 to 878 amino acids (aa) in length, with molecular weights ranging from 19.95 to 98.93 kDa and theoretical isoelectric points (pI) ranging from 4.64 to 9.43. Further analysis revealed that 23 SmCRK family members were identified in eggplant (*S. melongena*) (Table S3). The protein lengths ranged from 245 to 3193 aa, molecular weights ranged from 26.77 to 353.27 kDa, and theoretical pI values ranged from 5.37 to 9.41. Although SmCRK2 and SmCRK9 displayed considerably longer protein sequences than other SmCRK members, they were included in subsequent analyses based on the confirmation of their intact gene structures and conserved CRK-related domains through genome annotation and protein domain identification. In tomato (*S. lycopersicum*), 37 SlCRK family members were identified (Table S4), with protein lengths ranging from 93 to 862 aa, molecular weights ranging from 10.05 to 96.90 kDa, and theoretical pI ranging from 4.68 to 8.96.

**Figure 1:**
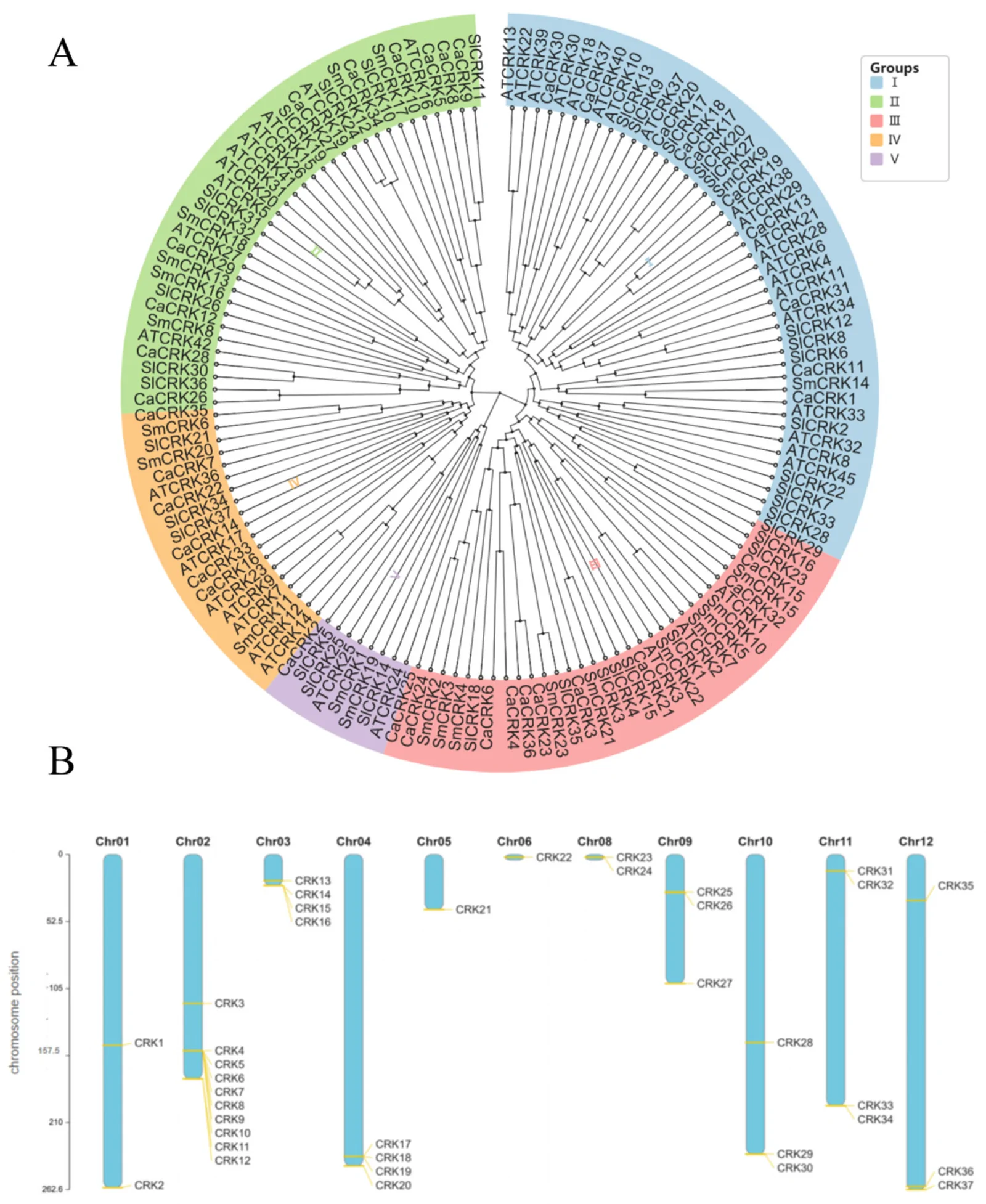
(**A**) The phylogenetic tree was constructed based on the full-length amino acid sequences of CRK proteins from *Capsicum annuum*, *Arabidopsis thaliana*, *Solanum lycopersicum*, and *Solanum melongena*. The phylogenetic tree was generated using MEGA 12. Different groups of CRK proteins were marked with different colors. (**B**) Physical distribution map of 37 *CaCRK* genes across the 12 chromosomes of *Capsicum annuum*.

To further investigate the evolutionary relationships of the CRK family among different plant species, CRK protein sequences from *A. thaliana*, *S. lycopersicum*, and *S. melongena* were selected together with CaCRK proteins to construct a phylogenetic tree (Figure 1A). Phylogenetic analysis of CRK proteins from four plant species revealed multiple clades, with several subgroups containing members from all four species, indicating their conserved origin. Among them, group III contained the largest number of members across all species and likely underwent prominent tandem duplication events. In contrast, groups IV and V exhibited species-specific distribution patterns, suggesting lineage-specific duplications in group IV and a Solanaceae-common ancestral origin followed by species retention in group V. Collectively, these results indicate that CRK family expansion in Solanaceae was driven by both ancient conservation and lineage-specific duplications. And in this study, particular attention was paid to the genetic characteristics of the pepper *CaCRK* family. Combined analysis of chromosomal localization information revealed that some CaCRK members located in the same chromosomal regions also exhibited close phylogenetic relationships (Figure 1B). For example, *CaCRK5*, *CaCRK6*, *CaCRK7*, *CaCRK8*, *CaCRK9*, and *CaCRK10* located on chromosome Chr02 were distributed in adjacent chromosomal regions and formed closely related branches in the phylogenetic tree. Similarly, *CaCRK17*, *CaCRK18*, and *CaCRK19* located on Chr04 showed a comparable clustering pattern. These results suggest that tandem duplication events may have contributed to the expansion of the *CaCRK* gene family and subsequently promoted functional diversification during long-term evolutionary processes.

### 3.2. Domain Architecture and Conserved Motif Analysis of CaCRK Proteins

**Figure 2:**
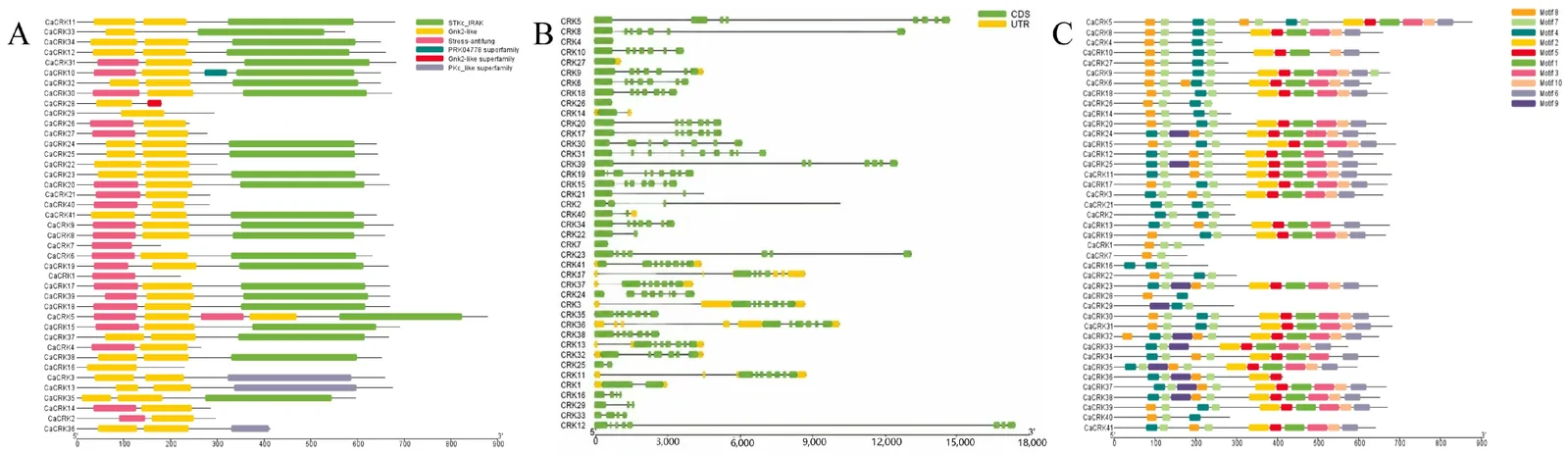
(**A**) Conserved domain architectures of CaCRK proteins. (**B**) Exon–intron structures of *CaCRK* genes. (**C**) Conserved motifs identified by MEME analysis in CaCRK proteins.

To further characterize the structural features and potential functional diversity of CaCRK proteins, domain prediction analysis was performed using the SMART and InterProScan databases. The results showed that CaCRK proteins exhibited highly conserved structural characteristics, with conserved DUF26 domains located at the N-terminus and a kinase domain located at the C-terminus (Figure 2A). Most CaCRK proteins contained two tandemly arranged DUF26 domains and one C-terminal kinase domain, which is consistent with the typical structural organization of CRK family proteins. However, several CaCRK members displayed distinct structural features. For example, CaCRK5 possessed a relatively long protein sequence and contained additional conserved regions, with an extended region remaining after the kinase domain. CaCRK36 and CaCRK3 also exhibited differences in domain organization compared with typical CRK proteins, showing varying degrees of domain loss or structural variation. In addition, differences in the number and arrangement of DUF26 domains were observed among some CaCRK proteins, suggesting that CaCRK family members may have undergone domain duplication, deletion, or recombination events during evolution, contributing to functional diversification.

To further investigate conserved regions within CaCRK protein sequences, conserved motif analysis was performed using MEME software (version 5.5.8) [34]. The results revealed that CaCRK family members contained multiple highly conserved motifs (Motif 1–Motif 10) (Figure 2C). Among them, Motif 1, Motif 2, and Motif 3 were widely distributed among most CaCRK members and were mainly located within the protein kinase domain, indicating that these motifs may contribute to the maintenance of kinase activity and structural stability. Meanwhile, the DUF26 domain regions also exhibited a high degree of sequence conservation, particularly at conserved sites enriched in cysteine residues. Most CaCRK members showed similar motif arrangement patterns, which were highly conserved among multiple CaCRK proteins, further supporting their common evolutionary origin. However, several members, including CaCRK5, CaCRK24, and CaCRK35, exhibited specific motif loss or additional motif occurrence, suggesting that these proteins may have undergone functional diversification during evolution.

### 3.3. Transcript Expression Patterns of CRK Genes Under Salt Stress

**Figure 3:**
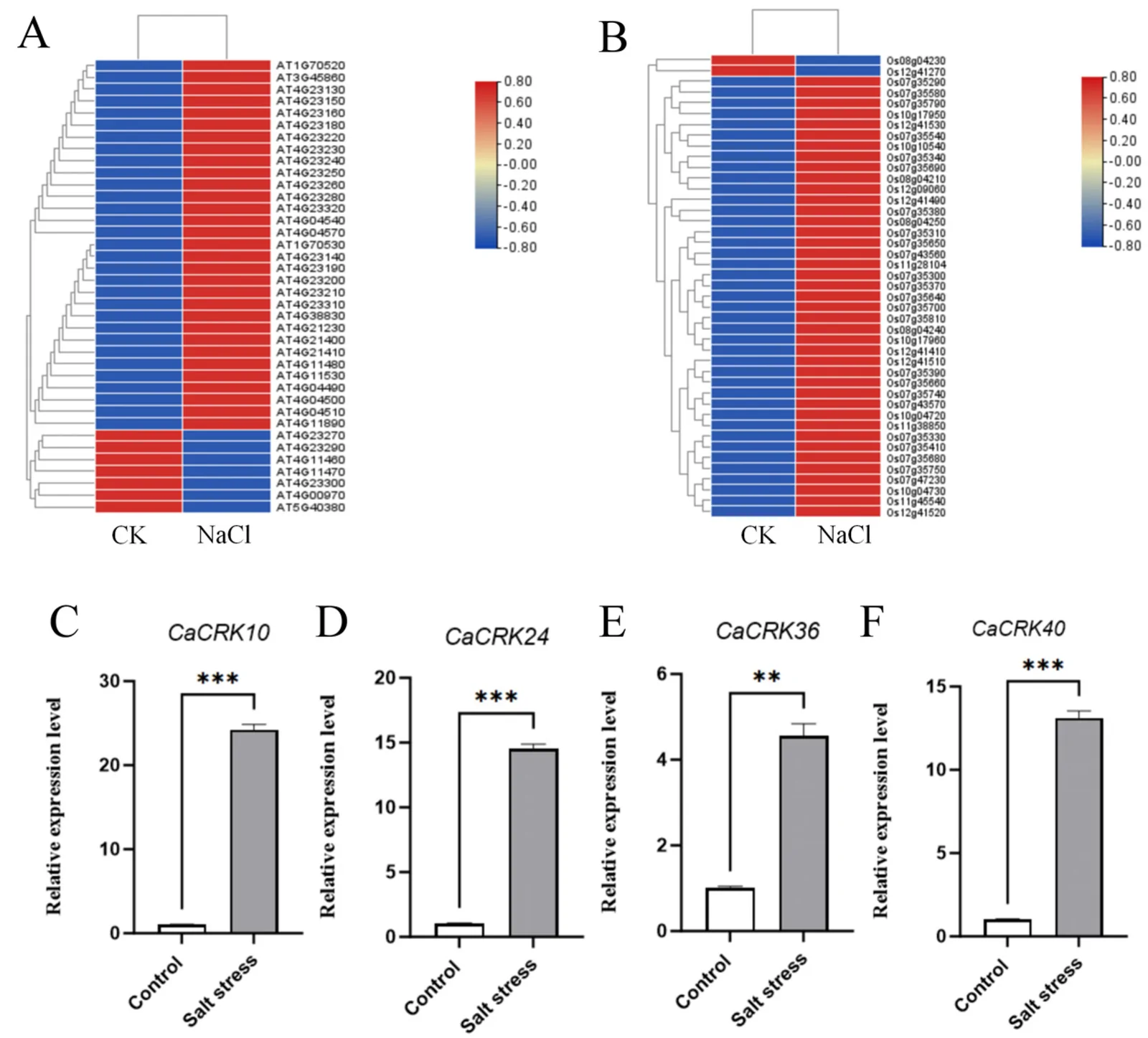
Expression heatmap of CRK family genes under salt stress. (**A**) Expression heatmap of CRK family genes in *Arabidopsis thaliana* under salt stress. (**B**) Expression heatmap of CRK family genes in rice under salt stress. (**C**–**F**) qRT-PCR analysis of four highly upregulated *CaCRK* genes (*CaCRK10*, *CaCRK24*, *CaCRK36*, and *CaCRK40*) compared with the control group. All four genes exhibited responses to salt stress. Statistical significance was defined as ** *p* < 0.01 and *** *p* < 0.001.

Based on publicly available transcriptome datasets from the NCBI Sequence Read Archive (SRA) database, the expression patterns of CRK family members in *Arabidopsis thaliana* and rice under salt stress conditions were further analyzed and visualized (Figure 3). The response characteristics of CRK genes under normal conditions (CK) and NaCl treatment were systematically evaluated through FPKM expression value normalization and hierarchical clustering analysis.

In *Arabidopsis* leaves, salt stress significantly affected the expression levels of multiple CRK genes. According to their expression patterns, *Arabidopsis* CRK members could be classified into two major response categories. A subset of CRK genes exhibited clear induction after NaCl treatment, including *At4G23220*, *At5G40380*, *At4G23190*, *At4G21230*, *At4G04570*, and *At4G23200*. These genes maintained relatively low expression levels under normal conditions but were significantly upregulated after salt treatment, suggesting that they may participate in salt stress perception and stress-responsive processes. In contrast, several CRK genes, including *At4G21340*, *At4G11530*, *At4G11890*, and *At1G70520*, showed obvious repression after salt treatment, indicating that CRK family members may undergo functional differentiation during salt stress responses.

Similarly, CRK genes in rice stem tissues displayed significant salt-responsive expression patterns. Following salt treatment, most *OsCRK* members showed increased expression trends. Among them, *Os08g04230*, *Os12g41270*, *Os07g35290*, *Os07g35580*, *Os07g35790*, and *Os10g17950* exhibited obvious upregulation under NaCl conditions. These salt-induced *OsCRK* genes may function as important regulatory factors involved in rice salt stress responses by participating in stress signal transduction, reactive oxygen species (ROS) homeostasis regulation, and cellular defense responses. In addition, some *OsCRK* members displayed decreased expression after salt treatment, suggesting the existence of salt-sensitive CRK members in rice.

Phylogenetic analysis based on CRK protein sequences revealed that *CaCRK10*, *CaCRK24*, *CaCRK36*, and *CaCRK40* clustered into the same phylogenetic clade with several salt-responsive CRK members from *Arabidopsis* and rice, showing relatively high sequence conservation. Therefore, these four *CaCRK* genes were selected for further expression analysis by qRT-PCR. Under salt stress treatment, all four *CaCRK* genes exhibited differential expression patterns. Among them, *CaCRK10* showed the strongest induction response, with transcript levels significantly increased under salt stress and reaching approximately 25-fold higher than those under control conditions. In addition, *CaCRK24*, *CaCRK36*, and *CaCRK40* also displayed varying degrees of upregulation, suggesting their potential roles in plant responses to environmental stress. Given the strong salt-responsive expression pattern of *CaCRK10*, it was selected as a candidate gene for subsequent functional characterization.

### 3.4. Structural Characteristics of CaCRK10 and Molecular Function Analysis Involved in Salt Stress Response

**Figure 4:**
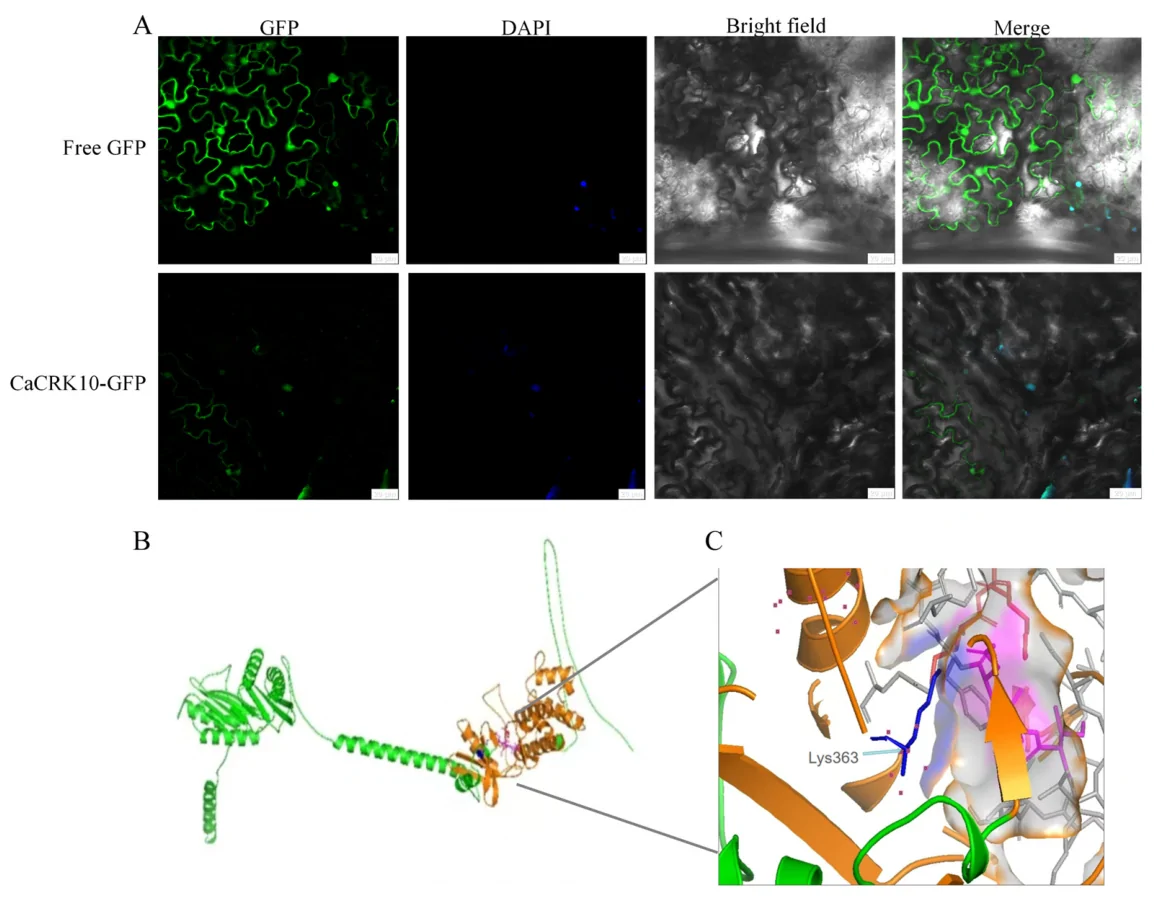
(**A**) The CDS sequence of *CaCRK10* was cloned into the pCAMBIA1300 vector and fused with GFP. The pCAMBIA1300-*CaCRK10* construct and GFP empty vector control were transformed into *Agrobacterium tumefaciens* strain GV3101 and subsequently infiltrated into tobacco cells for transient expression analysis. (**B**) Predicted three-dimensional structure and local structural characteristics of the CaCRK10 protein. (**C**) Enlarged view of the region containing residue Lys363, highlighting its local structural environment. The orange arrow indicates that the amino acids from Ala463 to Leu468 form a β-sheet structure.

To further determine the subcellular localization of CaCRK10, the coding sequence of *CaCRK10* was fused with green fluorescent protein (GFP) to generate the CaCRK10-GFP expression construct. The recombinant vector was transiently expressed in tobacco leaves using an Agrobacterium-mediated transformation method. Confocal laser scanning microscopy revealed that the fluorescence signal of the CaCRK10-GFP fusion protein was mainly localized to the plasma membrane region of tobacco cells, with a portion of the fluorescence signal detected in the nucleus. In contrast, the free GFP control protein exhibited widespread fluorescence distribution throughout the cytoplasm and nucleus (Figure 4A). Conserved domain analysis showed that CaCRK10 consists of 649 amino acids. The N-terminal region contains a signal peptide (1–28 aa), followed by an extracellular region containing two tandemly arranged DUF26 conserved domains (DUF26-1: 38–137 aa; DUF26-2: 153–240 aa), an extracellular linker region (241–270 aa), a transmembrane domain (approximately 271–299 aa), an intracellular serine/threonine protein kinase domain (approximately 341–600 aa), and a C-terminal tail (601–649 aa). (Figure 4B). These results indicate that CaCRK10 is primarily localized to the plant plasma membrane and may participate in stress-related signal transduction processes through its plasma membrane localization.

### 3.5. Functional Analysis of CaCRK10 Overexpression in Enhancing Salt Tolerance

**Figure 5:**
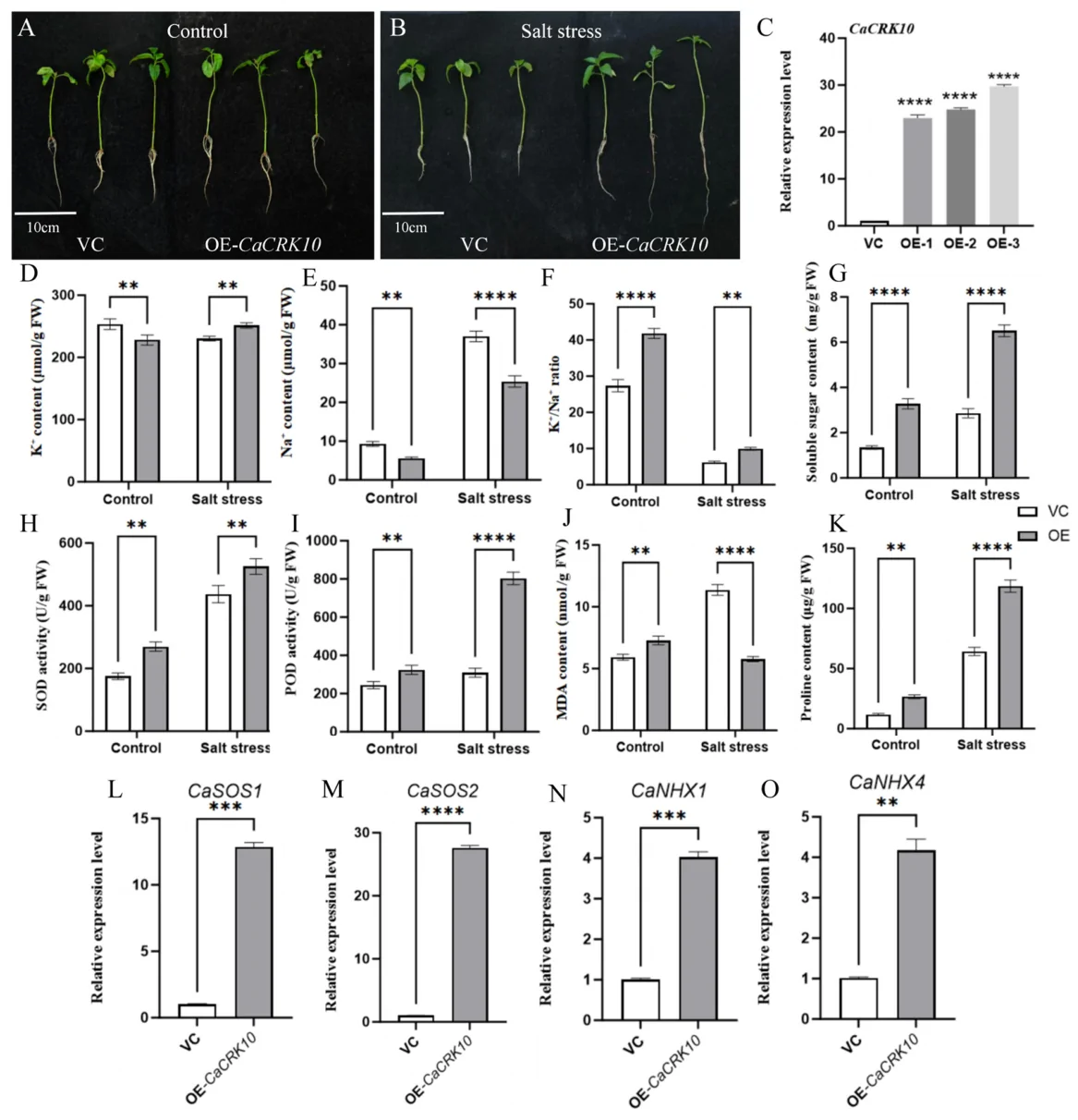
Physiological parameters were analyzed in empty vector control (VC) and *CaCRK10*-overexpressing (OE) transgenic pepper plants under control (CK) and salt stress (100 mM NaCl) conditions for 6 days. (**A**) Morphological phenotypes of plants under control conditions. (**B**) Morphological phenotypes of plants under NaCl treatment. (**C**) Relative expression levels of *CaCRK10* in VC and OE lines. (**D**) K^+^ content. (**E**) Na^+^ content. (**F**) K^+^/Na^+^ ratio. (**G**) Soluble sugar content. (**H**) SOD activity. (**I**) POD activity. (**J**) MDA content. (**K**) Proline content. Bars represent standard deviations (SD) from three independent biological replicates (*n* = 3). (**L**–**O**) Expression levels of salt-responsive marker genes in hairy roots of OE and VC composite plants after salt treatment. (**L**) *CaSOS1*. (**M**) *CaSOS2*. (**N**) *CaNHX1.* (**O**) *CaNHX4*. Data were obtained from three independent biological replicates (*n* = 3). Statistical significance was determined using a two-tailed Student’s *t*-test. ** *p* < 0.01, *** *p* < 0.001, and **** *p* < 0.0001.

To elucidate the role of *CaCRK10* in salt stress adaptation, transient overexpression composite pepper plants were first generated. *Agrobacterium rhizogenes* strain K599 harboring the *CaCRK10* overexpression construct or empty vector control was introduced into wounded stems of pepper seedlings to induce hairy root formation, and the infected plants were subsequently transferred into vermiculite for cultivation. Three positive overexpression lines were selected for functional analysis. RT-qPCR analysis was performed on OE-1 to OE-3 lines, and the results confirmed that *CaCRK10* transcript levels were significantly higher in OE lines than in vector control (VC) lines (Figure 5C). These *CaCRK10*-overexpressing (OE) composite pepper plants and empty vector control plants were subsequently subjected to functional characterization under salt stress conditions.

Phenotypic comparison under 100 mM NaCl treatment showed that OE plants exhibited significantly enhanced salt tolerance. Growth of VC plants was strongly inhibited, whereas OE plants maintained better overall growth performance under salt stress conditions (Figure 5A,B). To further investigate the physiological mechanisms underlying CaCRK10-mediated salt tolerance, ion homeostasis and stress-responsive physiological traits were analyzed. Compared with VC plants, *OE-CaCRK10* plants accumulated significantly higher levels of K^+^ and lower levels of Na^+^ under salt stress (Figure 5D,E), resulting in an increased K^+^/Na^+^ ratio (Figure 5F), indicating that *CaCRK10* overexpression contributed to maintaining cellular ion balance under saline conditions. In addition, OE plants exhibited significantly increased soluble sugar accumulation compared with VC plants under salt stress (Figure 5G), suggesting enhanced osmotic adjustment capacity. Furthermore, the activities of antioxidant enzymes, including superoxide dismutase (SOD) and peroxidase (POD), were significantly higher in OE plants than in VC plants (Figure 5H,I), indicating that *CaCRK10* overexpression enhanced ROS scavenging capacity and strengthened antioxidant defense. Consistently, *OE-CaCRK10* plants showed significantly lower malondialdehyde (MDA) content than VC plants under salt stress (Figure 5J), suggesting reduced membrane lipid peroxidation and oxidative damage. Moreover, OE plants accumulated higher levels of proline than VC plants (Figure 5K), further demonstrating improved osmotic regulation ability. Collectively, these results demonstrate that *CaCRK10* positively regulates salt tolerance in pepper by maintaining ion homeostasis, enhancing osmotic adjustment, activating antioxidant defense, and reducing oxidative damage, thereby promoting plant growth under salt stress conditions.

Furthermore, to explore the potential molecular mechanisms underlying CaCRK10-mediated salt tolerance, the expression patterns of several key salt-responsive genes were analyzed. Based on established salt stress signaling pathways, four candidate downstream genes were selected, including *CaSOS1* and *CaSOS2*, which are involved in SOS-mediated Na^+^ extrusion, and *CaNHX1* and *CaNHX4*, which function in vacuolar Na^+^/H^+^ exchange and ion compartmentalization. RT-qPCR analysis revealed that the transcript levels of *CaSOS1*, *CaSOS2*, *CaNHX1*, and *CaNHX4* were significantly higher in *CaCRK10*-overexpressing plants than in VC plants under salt stress conditions (Figure 5L–O). These findings indicate that *CaCRK10* may positively regulate salt tolerance by activating downstream ion homeostasis-related pathways, including SOS-dependent Na^+^ exclusion and NHX-mediated Na^+^ sequestration (Figure 5L–O).

### 3.6. Salt Stress Response Characteristics and Structural Functional Analysis of SmCRK6 in Eggplant

Based on CRK protein sequence alignment and phylogenetic analysis, *SmCRK1*, *SmCRK7*, *SmCRK6*, and *SmCRK10* were found to cluster into the same evolutionary branch with multiple salt-induced CRK members from *Arabidopsis thaliana* and rice, showing relatively high sequence conservation. qRT-PCR analysis revealed that all four *SmCRK* genes were induced to different degrees under salt treatment. Among them, *SmCRK6* exhibited the strongest response, with transcript levels increasing approximately 25-fold compared with the control group. *SmCRK1*, *SmCRK7*, and *SmCRK10* also showed significant salt-responsive expression patterns, suggesting that these genes may participate in plant adaptation to adverse environmental conditions.

**Figure 6:**
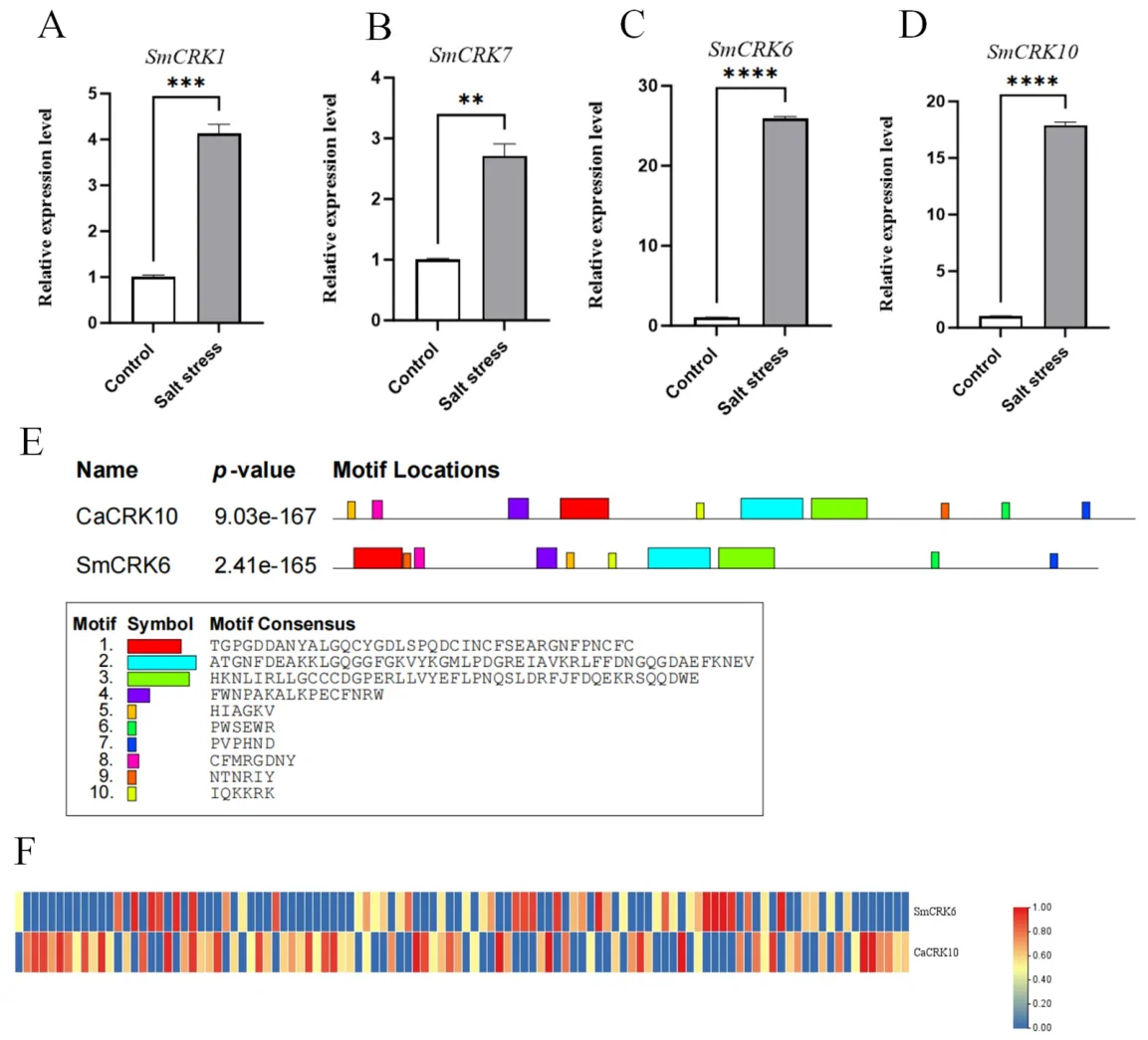
(**A**–**D**) Expression analysis of *SmCRK1*, *SmCRK7*, *SmCRK6*, and *SmCRK10* under salt stress conditions. Relative expression levels of these genes were determined by RT−qPCR after salt treatment for 0 h and 6 days. All tested genes exhibited responses to salt stress. Asterisks indicate statistical significance: ** *p* < 0.01, *** *p* < 0.001, and **** *p* < 0.0001. (**E**) Conserved motif distribution analysis of CaCRK10 and SmCRK6 proteins. (**F**) Heatmap analysis of amino acid sequence similarity between CaCRK10 and SmCRK6.

To further investigate the structural conservation and potential functional relationship between SmCRK6 and CaCRK10, conserved motifs within the two protein sequences were predicted and compared using the MEME online tool. The results showed that SmCRK6 and CaCRK10 shared multiple conserved motifs, and these motifs exhibited highly similar arrangement patterns and distribution positions along the protein sequences (Figure 6E), indicating a high degree of structural conservation between the two proteins. Potential phosphorylation sites of SmCRK6 and CaCRK10 proteins were further predicted using the NetPhos 3.1 online tool. Both proteins contained abundant potential phosphorylation sites, including Serine (Ser), Threonine (Thr), and Tyrosine (Tyr) residues, with similar distribution patterns (Figure S2). The presence of multiple potential regulatory phosphorylation sites in both proteins suggested that SmCRK6 and CaCRK10 may undergo similar post-translational modification regulation and participate in plant salt stress responses through conserved signaling mechanisms. Furthermore, sequence similarity heatmap analysis revealed a high degree of amino acid conservation between SmCRK6 and CaCRK10 across multiple regions, particularly within functionally relevant domains (Figure 6F). These results suggest that SmCRK6 and CaCRK10 may have undergone conserved evolutionary processes and retained similar structural characteristics, providing a potential structural basis for their functional association in salt stress responses.

**Figure 7:**
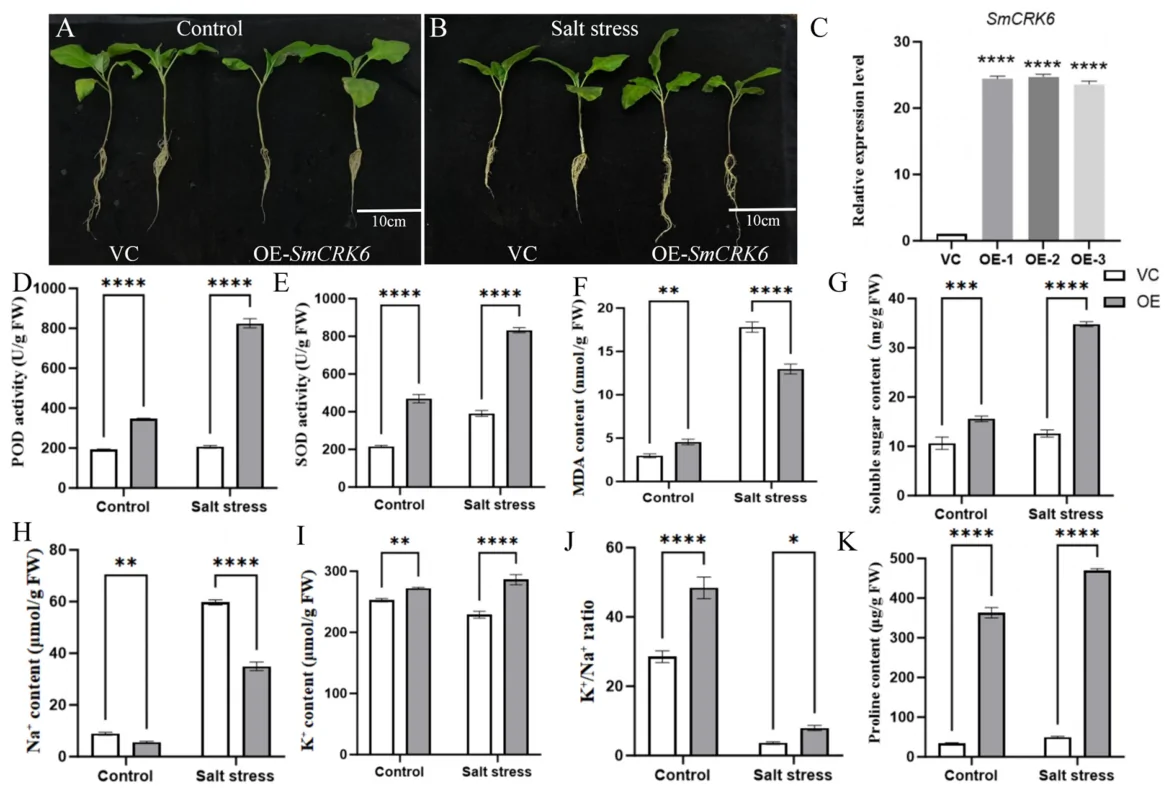
Physiological parameters were analyzed in empty vector control (VC) and *SmCRK6*-overexpressing (OE) transgenic eggplant plants under control (CK) and salt stress (100 mM NaCl) conditions for 6 days. (**A**) Morphological phenotypes of plants under control conditions. (**B**) Morphological phenotypes of plants under NaCl treatment conditions. (**C**) Relative expression levels of *SmCRK6* in VC and OE lines. (**D**–**K**) Physiological parameters of VC and *SmCRK6*-overexpressing plants under salt stress conditions, including POD activity, SOD activity, MDA content, soluble sugar content, Na^+^ content, K^+^ content, K^+^/Na^+^ ratio and proline content. Bars represent standard deviations (SD) from three independent biological replicates (*n* = 3). Asterisks indicate significant differences: * *p* < 0.05, ** *p* < 0.01, *** *p* < 0.001, and **** *p* < 0.0001.

To investigate the role of *SmCRK6* in salt stress tolerance, composite eggplant plants with transient *SmCRK6* overexpression were generated using *Agrobacterium rhizogenes* strain K599-mediated hairy root transformation. Three independent overexpression lines (OE-1 to OE-3) were selected, and RT-qPCR analysis confirmed significantly higher *SmCRK6* expression levels in OE lines compared with vector control (VC) plants (Figure 7C). Under 100 mM NaCl treatment, *SmCRK6*-overexpressing plants exhibited enhanced salt tolerance, with reduced growth inhibition, longer roots, greener leaves, and improved overall growth compared with VC plants (Figure 7A,B). Physiological analyses further revealed that *SmCRK6* overexpression enhanced antioxidant capacity and improved salt stress adaptation. Compared with VC plants, OE plants showed enhanced antioxidant enzyme activities, including POD and SOD, under both control and salt stress conditions. Notably, OE plants accumulated significantly lower levels of MDA under salt stress. (Figure 7D–F), indicating enhanced ROS scavenging capacity and reduced oxidative damage. In addition, OE plants accumulated higher levels of soluble sugars and proline under salt stress (Figure 7G,K), suggesting improved osmotic adjustment. Furthermore, *SmCRK6* overexpression improved ion homeostasis by reducing Na^+^ accumulation, increasing K^+^ content, and maintaining a higher K^+^/Na^+^ ratio under salt stress (Figure 7H–J). Collectively, these results demonstrate that *SmCRK6* enhances salt tolerance in eggplant by strengthening antioxidant defense, regulating ion balance, and promoting osmotic adjustment.

## 4. Discussion

Plant receptor-like kinases (RLKs) serve as important membrane-localized signal perception components and play essential roles in plant responses to environmental stresses [35]. CRK proteins have been implicated in salt, drought, and oxidative stress responses in several plant species. These studies have mainly examined individual CRK genes, whereas information on the CRK family in horticultural Solanaceae crops is still relatively limited. In particular, the contribution of CRK genes to salt tolerance in pepper and eggplant has not been systematically investigated. In this study, we systematically identified the CRK family members in *Capsicum annuum* L. and integrated phylogenetic analysis, expression profiling, and genetic transformation experiments to demonstrate the critical roles of CRK genes in salt stress adaptation in Solanaceae crops. Our results revealed that the *CaCRK* family has maintained high structural conservation during evolution [17]. Most CaCRK proteins contain the typical extracellular DUF26 domains, transmembrane regions, and intracellular serine/threonine kinase domains [36], suggesting that they may perceive extracellular environmental stimuli and subsequently activate downstream signaling pathways involved in stress regulation [37,38].

In the present study, *CaCRK10* and *SmCRK6* exhibited significant induction under salt stress conditions, indicating that these two genes may participate in salt stress perception and response. This observation is consistent with the functional characteristics of CRK proteins as membrane-associated receptor-like kinases involved in environmental signal transduction. Overexpression analysis also demonstrated that *CaCRK10* and *SmCRK6* enhanced salt tolerance in pepper and eggplant plants, respectively. The improved salt tolerance was mainly associated with reduced membrane lipid peroxidation, enhanced antioxidant enzyme activities, and improved K^+^/Na^+^ homeostasis. These findings suggest that *CaCRK10* and *SmCRK6* may contribute to the establishment of a salt tolerance regulatory network involving ROS signaling, ion homeostasis, and osmotic adjustment [39].

The study focused on CRK members from two economically important Solanaceae crops, pepper and eggplant. Based on genome-wide identification and salt-responsive expression analysis, *CaCRK10* and *SmCRK6* showed relatively high expression levels and strong responses to salt stress in pepper and eggplant, respectively. Interestingly, several salt stress-related physiological indicators were already altered under normal growth conditions, suggesting that *CaCRK10* and *SmCRK6* may possess basal regulatory functions rather than acting solely as stress-inducible genes. These findings indicate that these two genes may represent important CRK candidates involved in salt adaptation in different *Solanaceous* species.

Although pepper and eggplant have undergone distinct evolutionary trajectories, both *CaCRK10* and *SmCRK6* contribute to salt stress responses, suggesting conserved functions of CRK proteins in stress adaptation. However, species-specific evolutionary processes may have resulted in differences in their regulatory mechanisms. Both genes were found to enhance plant salt tolerance, although they differed in their responses to salt stress and in the downstream pathways that may contribute to this effect. These variations may reflect the unique regulatory strategies developed by different *Solanaceous* species during long-term environmental adaptation. Therefore, the comparative investigation of *CaCRK10* and *SmCRK6* does not simply aim to evaluate functional superiority between the two genes, but rather to elucidate conserved and species-specific regulatory mechanisms of CRK members in Solanaceae crops, thereby advancing our understanding of RLK-mediated salt stress signaling pathways.

Furthermore, we found that overexpression of *CaCRK10* significantly enhanced the expression levels of several SOS- and NHX-related genes involved in ion homeostasis regulation, suggesting that *CaCRK10* may participate in maintaining ion balance during salt stress responses. This result provides an additional mechanistic perspective beyond the previously described associations of CRKs with ROS-related stress responses, by linking *CaCRK10* activity with transcriptional changes in genes involved in Na^+^ exclusion and sequestration. Plants have evolved multiple regulatory mechanisms to reduce cytotoxic Na^+^ accumulation to avoid excessive Na^+^ in plant cells, which causes cytosolic ion imbalance and metabolic disruption. The SOS pathway represents a major regulatory module involved in salt stress adaptation by promoting Na^+^ extrusion from the cytoplasm to alleviate Na^+^ toxicity [40]. Meanwhile, NHX family proteins mediate Na^+^/H^+^ exchange and facilitate Na^+^ sequestration into vacuoles, thereby maintaining intracellular ion homeostasis [41]. In this study, *CaCRK10* overexpression promoted the expression of key SOS and NHX genes, indicating that *CaCRK10* may function as an upstream signaling regulator to enhance salt tolerance by regulating SOS-mediated Na^+^ exclusion and NHX-mediated vacuolar Na^+^ compartmentalization.

As membrane-localized receptor-like kinases, CRK proteins are generally involved in extracellular signal perception, protein phosphorylation, and downstream signal transduction rather than directly regulating gene transcription [18]. It remains unclear whether CaCRK10 directly phosphorylates key components of the SOS/NHX pathways or indirectly affects the expression of ion homeostasis-related genes through other signaling modules. Previous studies have demonstrated that plant RLKs can convert extracellular environmental signals into intracellular defense responses by recruiting downstream kinases, modulating Ca^2+^ signaling, and activating MAPK cascades [42]. Thus, CaCRK10 and SmCRK6 proteins may function through similar signaling networks, transmitting salt stress perception signals to ROS homeostasis pathways and ion transport regulatory modules, ultimately enhancing plant salt tolerance [43].

Although this study provides preliminary evidence supporting the roles of pepper and eggplant CRK genes in salt stress responses, the phosphorylation mechanisms and downstream targets of *CaCRK10* and *SmCRK6* remain to be elucidated. Future studies integrating protein–protein interaction analysis [44], phosphoproteomic approaches, and CRISPR/Cas9-mediated gene editing technology [45] will help identify interacting proteins and direct targets of CaCRK10, thereby determining whether CaCRK10 connects upstream receptor perception with downstream SOS/NHX-mediated ion homeostasis through specific kinase cascades or MAPK signaling modules [46]. In addition, the generation of *CaCRK10* loss-of-function mutants combined with multi-omics analyses will facilitate the construction of a more comprehensive CRK-mediated salt stress regulatory network. These studies will further elucidate the molecular mechanisms underlying RLK-mediated salt adaptation in Solanaceae crops and provide valuable genetic resources for improving crop salt tolerance through CRK-based breeding strategies.

This study extends previous investigations of CRK-mediated abiotic stress responses by integrating genome-wide CRK characterization with cross-species functional validation in pepper and eggplant. The results identify *CaCRK10* and *SmCRK6* as positive regulators of salt tolerance and associate their functions with antioxidant defense, ROS damage limitation, osmotic adjustment, and ion homeostasis. The identification of similar salt-tolerance phenotypes in two Solanaceae crops, together with the association of *CaCRK10* with SOS- and NHX-related ion homeostasis pathways, provides new evidence for the potential conservation of CRK-mediated salt adaptation in horticultural crops. These findings provide candidate genetic resources for further investigation and improvement of salt tolerance in Solanaceae crops.

## 5. Conclusions

This study performed a comprehensive analysis of the CRK gene family in two economically important Solanaceae crops, revealing the conserved structural characteristics and evolutionary relationships of CRK family members. A total of 37 CRK family members were identified in pepper, and multiple CRK genes exhibited significant transcriptional responses under salt stress, suggesting their potential involvement in plant adaptation to environmental stresses. Functional validation demonstrated that CaCRK10 in pepper and its eggplant homolog SmCRK6 act as positive regulators of salt stress tolerance. Overexpression of *CaCRK10* and *SmCRK6* significantly enhanced plant growth performance under salt stress conditions. The underlying mechanisms mainly involved enhanced antioxidant defense capacity, reduced ROS (reactive oxygen species)-induced oxidative damage, maintenance of K^+^/Na^+^ ion homeostasis, and promotion of osmotic adjustment. In addition, CaCRK10 promoted the expression of SOS- and NHX-related ion regulatory genes, indicating that it may participate in coordinating ion transport processes and salt stress signaling pathways. Comparative analysis of CaCRK10 and SmCRK6 further revealed conserved protein structural characteristics and similar regulatory features, supporting the notion that CRK family members possess conserved functions in salt stress adaptation in Solanaceae plants. Collectively, this study expands our understanding of CRK-mediated stress signaling mechanisms in Solanaceae horticultural crops and provides valuable candidate gene resources and theoretical foundations for the development of salt-tolerant Solanaceae varieties.

## Data Availability

The original contributions presented in this study are included in the article and Supplementary Material. Further inquiries can be directed to the corresponding authors.

## Supplementary Materials

The following supporting information can be downloaded at: https://www.mdpi.com/article/10.3390/cimb48090965/s1.

## Abbreviations

The following abbreviations are used in this manuscript:

CRK	Cysteine-rich receptor-like kinases
MDA	Malondialdehyde
SOD	Superoxide dismutase
POD	Peroxidase
ROS	Reactive oxygen species
HMM	Hidden Markov Model
CDD	Conserved Domain Database
pI	Isoelectric point
NJ	Neighbor-Joining
MEME	Multiple EM for Motif Elicitation
MCScanX	Multiple Collinearity Scan toolkit
TBtools	Toolkit for Biologists
FPKM	Fragments Per Kilobase of transcript per Million mapped reads
SOS	Salt Overly Sensitive
NHX	Na^+^/H^+^ exchanger
