# Supplementary material for: Genome-Wide Identification of CRK Family Members in Solanaceae Crops and Functional Characterization of CaCRK10 and SmCRK6 in Salt Stress Response
Source: Curr Issues Mol Biol. 2026 Sep 21;48(9):965. doi: 10.3390/cimb48090965 (PMC13605080; doi:10.3390/cimb48090965)
Supplement: Supplementary file 1 [file cimb-48-00965-s001.zip › Supplementary Materials Figure S1.pdf]

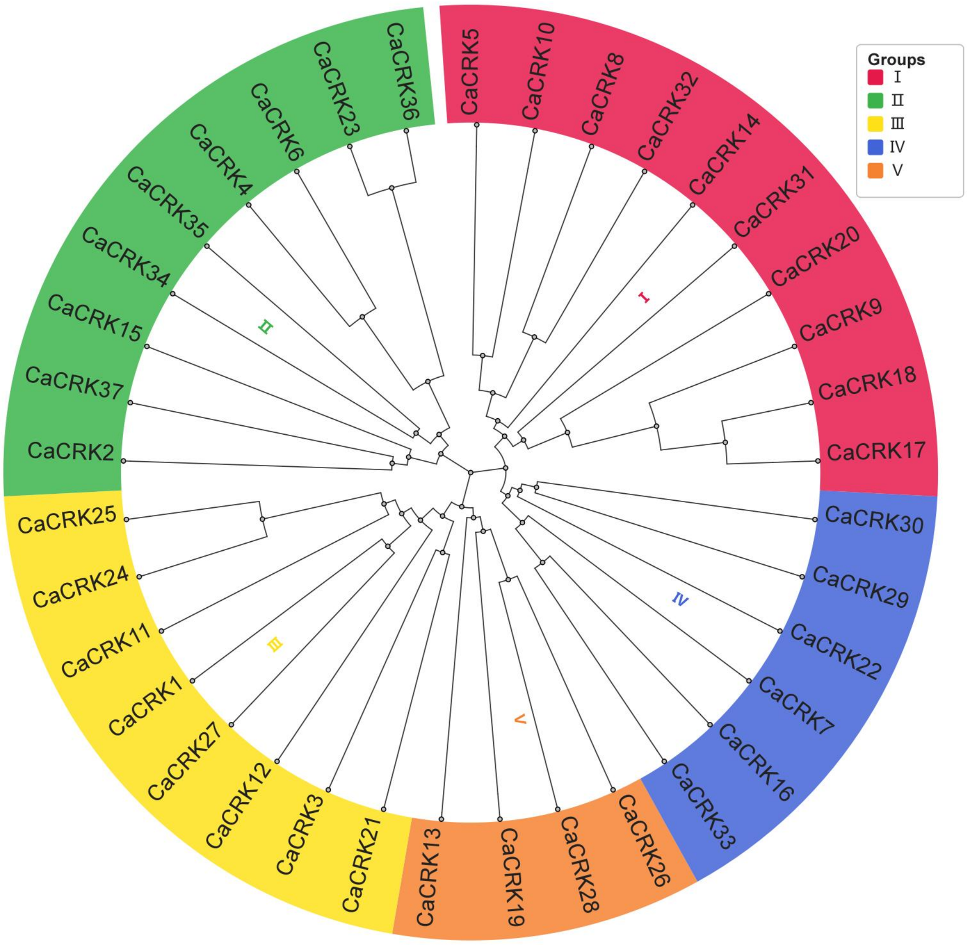

Figure S1 Phylogenetic analysis and classification of the CaCRK protein family in *Capsicum annuum*.
