# Supplementary material for: Genome-Wide Identification of CRK Family Members in Solanaceae Crops and Functional Characterization of CaCRK10 and SmCRK6 in Salt Stress Response
Source: Curr Issues Mol Biol. 2026 Sep 21;48(9):965. doi: 10.3390/cimb48090965 (PMC13605080; doi:10.3390/cimb48090965)
Supplement: Supplementary file 1 [file cimb-48-00965-s001.zip › Supplementary Materials Figure S2.pdf]

A CaCRK10

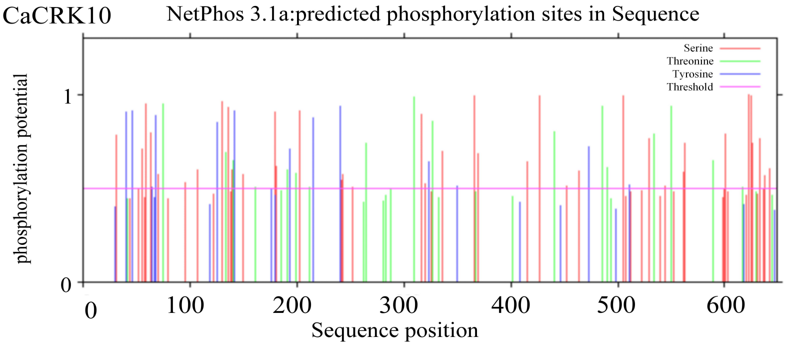

B SmCRK6

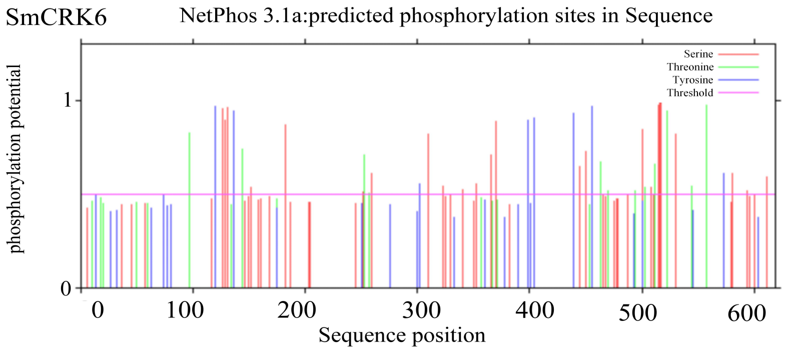

Figure S2 Prediction analysis of phosphorylation sites in CaCRK10 (A) and SmCRK6 (B) proteins. Potential phosphorylation sites of serine (Ser), threonine (Thr), and tyrosine (Tyr) residues were predicted using NetPhos 3. 1. Different colored bars represent the predicted phosphorylation probabilities of different amino acid residues, and the horizontal line indicates the prediction threshold.
