# Supplementary material for: Genome-Wide Identification of CRK Family Members in Solanaceae Crops and Functional Characterization of CaCRK10 and SmCRK6 in Salt Stress Response
Source: Curr Issues Mol Biol. 2026 Sep 21;48(9):965. doi: 10.3390/cimb48090965 (PMC13605080; doi:10.3390/cimb48090965)
Supplement: Supplementary file 1 [file cimb-48-00965-s001.zip › Supplementary Materials Table S1.pdf]

Supplementary Table S1. Primers used in this study		
Primer Name	Primer Sequence	Purposes
SmActin-F	GTCGGAATGGGACAGAAGGATG	Primers for qRT-PCR
SmActin-R	GTGCCTCAGTCAGGAGAACAGGGT	
SmTubulin-F	AGCAATTGGTCTTTTGGAGGGTG	
SmTubulin-R	AAGGAGAGTTCCCATTCCTGATCC	
CaActin-F	TGTTGGACTCTGGTGATGGTGTG	
CaActin-R	CCGTTCAGCCGTGGTGGTG	
CaTubulin-F	GAGGGTGAGTGAGCAGTTC	
CaTubulin-R	CTTCATCGTCATCTGCTGTC	
CaCRK10-qPCR-F	CACCCGTGTAGCTGGTACAA	
CaCRK10-qPCR-R	TGAGGGTCTGATCTCCGTGT	
CaCRK40-qPCR-F	AGGGGGAATTGCATCTGGTG	
CaCRK40-qPCR-R	CTTGCTTTGAGATCGCGGTG	
CaCRK36-qPCR-F	CTCAGCATAGCACAGCCTGA	
CaCRK36-qPCR-R	AAAAATCAGGGGCCTGCGTA	
CaCRK24-qPCR-F	TAGCAAGTGAGCAGATCCCG	
CaCRK24-qPCR-R	GCCTTGGTCTTCGTTCCACT	
SmCRK1-qPCR-F	CGGCCATGCACTGCAATTTT	
SmCRK1-qPCR-R	CAGCCATCACCATCACCACT	
SmCRK7-qPCR-F	AAACGCTGGAGACACACCAA	
SmCRK7-qPCR-R	TCGTGACTAGGTCCTGCAGA	
SmCRK6-qPCR-F	GGCTAGGCTGTTTCCAGAGG	
SmCRK6-qPCR-R	AAACTACTGCCTCCGGAAGC	
SmCRK10-qPCR-F	AGGGGCAATTGAGTGATGGG	
SmCRK10-qPCR-R	TTCAGGGTAGAGTCGAGCCA	
CaSOS2-qPCR-F	CTCACTGTCACAGCAAGGGT	
CaSOS2-qPCR-R	CATTGAACACAGCACGCACA	
CaSOS1-qPCR-F	CGTTTTGCTCGTCATTGGCA	
CaSOS1-qPCR-R	GGCCAATCACGACAGATCCA	
CaNHX1-qPCR-F	CGTTAACGCGTTAGCTGACG	
CaNHX1-qPCR-R	TCTCAACCAACCGCGAATCA	
CaNHX4-qPCR-F	GGTGTCCTTTCTCGGCAGAT	
CaNHX4-qPCR-R	TGAAGCCCAAACTCGAGCAT	
pEasy-CaCRK10-F	[illegible]CGTTAATTAAATGACCAAACCCAAAAAATTC[illegible]	Primers for cloning
pEasy-CaCRK10-R	[illegible]CGGGCGCGCCCTAATCTCTCACAGGACGTTT[illegible]	
pEasy-SmCRK6-F	ATGCATTCTTCCTTGATGTT	
pEasy-SmCRK6-R	TCACCTGCCTTCATTTATAGA	
CaCRK10-1300GFP-F	ATGACCAAACCCAAAAAATTGCT	Primers for subcellular localization
CaCRK10-1300GFP-R	ATCTCTCACAGGACGTTTTCCAT	
