# Supplementary material for: Genome-Wide Identification of CRK Family Members in Solanaceae Crops and Functional Characterization of CaCRK10 and SmCRK6 in Salt Stress Response
Source: Curr Issues Mol Biol. 2026 Sep 21;48(9):965. doi: 10.3390/cimb48090965 (PMC13605080; doi:10.3390/cimb48090965)
Supplement: Supplementary file 1 [file cimb-48-00965-s001.zip › Supplementary Materials Table S2.pdf]

Supplementary Table S2. Physicochemical Characteristics of CaCRK Proteins

Name	Gene ID	Amino Acids	Theoretical pI	Molecular weight	Instability index	GRAVY	Subcellular Location
CaCRK1	ZLC01G0023650	221	8.68	24.94	48.94	-0.252	Plasma membrane
CaCRK2	ZLC01G0037030	296	6.36	32.24	41.1	0.085	Plasma membrane
CaCRK3	ZLC02G0008240	659	6.02	74.17	43.49	-0.149	Plasma membrane
CaCRK4	ZLC02G0020610	265	6.2	29.44	42.35	-0.317	Plasma membrane
CaCRK5	ZLC02G0020620	878	7.03	98.93	41.64	-0.261	Plasma membrane
CaCRK6	ZLC02G0020630	631	6.65	70.62	40.61	-0.176	Plasma membrane
CaCRK7	ZLC02G0020650	179	6.23	19.95	59.41	-0.355	Plasma membrane
CaCRK8	ZLC02G0020660	659	7.43	73.6	50.88	-0.205	Plasma membrane
CaCRK9	ZLC02G0020670	677	7.14	75.7	42.8	-0.183	Plasma membrane
CaCRK10	ZLC02G0020680	649	7.43	72.46	42.02	-0.258	Plasma membrane
CaCRK11	ZLC02G0035370	680	8.09	75.1	41.18	-0.138	Plasma membrane
CaCRK12	ZLC02G0035410	660	8.78	72.8	29.14	-0.149	Plasma membrane
CaCRK13	ZLC03G0012460	675	7.15	75.12	50.31	-0.152	Plasma membrane
CaCRK14	ZLC03G0014170	286	5.7	31.57	38.93	-0.073	Plasma membrane
CaCRK15	ZLC03G0014190	691	7.18	77.21	44.05	-0.136	Plasma membrane
CaCRK16	ZLC03G0014210	230	8.12	25.8	43.93	-0.314	Plasma membrane
CaCRK17	ZLC04G0025290	670	6.03	74.3	50.04	-0.162	Plasma membrane
CaCRK18	ZLC04G0025300	670	5.75	74.57	47.86	-0.109	Plasma membrane
CaCRK19	ZLC04G0025310	666	5.44	73.46	49.37	-0.093	Plasma membrane
CaCRK20	ZLC04G0026540	668	6.16	74.24	49.19	-0.159	Plasma membrane
CaCRK21	ZLC05G0010560	284	7.86	30.63	35.88	0.12	Plasma membrane
CaCRK22	ZLC06G0001740	300	8.88	32.11	37.09	-0.055	Plasma membrane
CaCRK23	ZLC08G0000780	647	8.45	71.77	36.18	-0.162	Plasma membrane
CaCRK24	ZLC08G0000790	644	8.9	71.56	32.2	-0.12	Plasma membrane
CaCRK25	ZLC09G0007520	240	6.71	26.83	37.95	-0.331	Plasma membrane
CaCRK26	ZLC09G0007560	279	4.64	31.01	43.72	-0.14	Plasma membrane
CaCRK27	ZLC09G0011420	181	8.83	20.03	24.25	0.012	Plasma membrane
CaCRK28	ZLC10G0010690	294	9.43	32.26	45.5	0.185	Plasma membrane
CaCRK29	ZLC10G0020430	674	8.92	76.76	43.04	-0.232	Plasma membrane
CaCRK30	ZLC10G0020440	682	6.29	75.75	46.38	-0.176	Plasma membrane
CaCRK31	ZLC11G0005600	649	8.21	72.17	35.75	-0.103	Plasma membrane
CaCRK32	ZLC11G0005610	574	8.15	64.31	35.36	-0.139	Plasma membrane
CaCRK33	ZLC11G0014510	649	8.86	71.9	31.8	-0.104	Plasma membrane
CaCRK34	ZLC11G0014520	596	8.5	66.5	43.02	-0.31	Plasma membrane
CaCRK35	ZLC12G0009610	670	7.82	75.96	45.61	-0.102	Plasma membrane
CaCRK36	ZLC12G0027880	283	6.85	30.83	42.01	-0.002	Plasma membrane
CaCRK37	ZLC12G0029220	641	8.64	70.86	39.2	-0.137	Plasma membrane
