# Supplementary material for: Genome-Wide Identification of CRK Family Members in Solanaceae Crops and Functional Characterization of CaCRK10 and SmCRK6 in Salt Stress Response
Source: Curr Issues Mol Biol. 2026 Sep 21;48(9):965. doi: 10.3390/cimb48090965 (PMC13605080; doi:10.3390/cimb48090965)
Supplement: Supplementary file 1 [file cimb-48-00965-s001.zip › Supplementary Materials Table S3.pdf]

Supplementary Table S3. Physicochemical Characteristics of SmCRK Proteins

Name	Gene ID	Amino Acids	Theoretical pI	Molecular weight	Instability index	GRAVY	Subcellular Location
SmCRK1	SMEL4.1_01g028820	248	9.41	27.45	38.68	-0.129	Plasma membrane
SmCRK2	SMEL4.1_01g037970	3193	8.44	353.27	35.54	-0.099	Plasma membrane
SmCRK3	SMEL4.1_01g037980	634	8.48	68.95	34.96	0.029	Plasma membrane
SmCRK4	SMEL4.1_01g037990	768	9.29	84.09	34.45	0.019	Plasma membrane
SmCRK5	SMEL4.1_01g038000	380	8.58	41.58	32.42	-0.022	Plasma membrane
SmCRK6	SMEL4.1_01g038010	619	8.32	68.55	42.06	-0.235	Plasma membrane
SmCRK7	SMEL4.1_01g038020	655	8.77	72.91	37.14	-0.15	Plasma membrane
SmCRK8	SMEL4.1_02g010130	610	6.05	68.33	38.96	-0.133	Plasma membrane
SmCRK9	SMEL4.1_02g018140	2762	6.92	308.91	45.83	-0.259	Plasma membrane
SmCRK10	SMEL4.1_02g029890	665	8.58	72.71	29.01	-0.079	Plasma membrane
SmCRK11	SMEL4.1_03g002120	545	6.29	60.44	45.75	-0.056	Plasma membrane
SmCRK12	SMEL4.1_03g010430	649	8.9	72.05	38.04	-0.127	Plasma membrane
SmCRK13	SMEL4.1_03g010450	661	8.22	73.89	43.23	-0.22	Plasma membrane
SmCRK14	SMEL4.1_03g022100	275	8.61	31.09	43.31	-0.142	Plasma membrane
SmCRK15	SMEL4.1_03g024630	671	8.32	74.75	45.68	-0.097	Plasma membrane
SmCRK16	SMEL4.1_05g007440	245	5.37	26.77	37.28	-0.176	Plasma membrane
SmCRK17	SMEL4.1_05g013780	364	9.36	40.53	30.74	-0.034	Plasma membrane
SmCRK18	SMEL4.1_05g017580	733	6.11	84.04	52.67	-0.274	Plasma membrane
SmCRK19	SMEL4.1_08g012040	248	8.63	27.51	39.81	-0.206	Plasma membrane
SmCRK20	SMEL4.1_11g011170	493	8.32	55.27	34.36	-0.506	Plasma membrane
SmCRK21	SMEL4.1_12g013820	246	8.25	27.43	50.42	-0.297	Plasma membrane
SmCRK22	SMEL4.1_00g012050	639	8.35	71.13	41.92	-0.183	Plasma membrane
SmCRK23	SMEL4.1_00g012060	642	8.47	70.95	40.83	-0.184	Plasma membrane
