# Supplementary material for: Genome-Wide Identification of CRK Family Members in Solanaceae Crops and Functional Characterization of CaCRK10 and SmCRK6 in Salt Stress Response
Source: Curr Issues Mol Biol. 2026 Sep 21;48(9):965. doi: 10.3390/cimb48090965 (PMC13605080; doi:10.3390/cimb48090965)
Supplement: Supplementary file 1 [file cimb-48-00965-s001.zip › Supplementary Materials Table S4.pdf]

Supplementary Table S4. Physicochemical Characteristics of SlCRK Proteins

Name	Gene ID	Amino Acids	Theoretical pI	Molecular weight	Instability index	GRAVY	Subcellular Location
SlCRK1	Solyc01g007960	651	7.49	72.16	33.65	-0.125	Plasma membrane
SlCRK2	Solyc01g007970	647	8.33	71.84	32.47	-0.171	Plasma membrane
SlCRK3	Solyc01g007980	638	8.19	71.18	45.24	-0.142	Plasma membrane
SlCRK4	Solyc01g007990	638	8.05	70.71	34.27	-0.152	Plasma membrane
SlCRK5	Solyc01g091040	299	5.42	32.57	33.19	0.049	Plasma membrane
SlCRK6	Solyc02g067770	137	4.88	15.4	39.4	0.201	Plasma membrane
SlCRK7	Solyc02g067780	495	6.07	55.95	43.11	-0.196	Plasma membrane
SlCRK8	Solyc02g079990	666	6.52	74.18	43.88	-0.263	Plasma membrane
SlCRK9	Solyc02g080010	862	6.89	96.9	51.7	-0.289	Plasma membrane
SlCRK10	Solyc02g080050	153	4.86	17.34	53.51	-0.296	Plasma membrane
SlCRK11	Solyc02g080060	367	8.81	41.31	39.35	-0.132	Plasma membrane
SlCRK12	Solyc02g080070	676	6.16	75.44	43.15	-0.156	Plasma membrane
SlCRK13	Solyc02g080080	671	6.02	74.62	34.16	-0.112	Plasma membrane
SlCRK14	Solyc02g080090	391	6.27	43.27	34.13	-0.219	Plasma membrane
SlCRK15	Solyc02g086590	668	8.56	73.28	30.85	-0.138	Plasma membrane
SlCRK16	Solyc03g005690	300	8.58	32.04	36.6	-0.011	Plasma membrane
SlCRK17	Solyc03g031550	150	6.11	17.16	21.59	0.181	Plasma membrane
SlCRK18	Solyc03g111520	93	7.66	10.05	42.58	-0.913	Plasma membrane
SlCRK19	Solyc03g111530	326	8.15	37.29	43.84	-0.325	Plasma membrane
SlCRK20	Solyc03g111540	675	6.17	75.89	43.52	-0.114	Plasma membrane
SlCRK21	Solyc03g112730	277	5.75	30.36	31.84	-0.069	Plasma membrane
SlCRK22	Solyc04g007950	320	8.3	35.19	48.31	-0.084	Plasma membrane
SlCRK23	Solyc04g026140	138	4.68	15.31	32.03	0.052	Plasma membrane
SlCRK24	Solyc05g005050	299	8.8	32.85	29.36	0.054	Plasma membrane
SlCRK25	Solyc05g005070	719	6.81	80.57	42.39	-0.103	Plasma membrane
SlCRK26	Solyc05g018930	184	5.29	20.24	37.68	0.091	Plasma membrane
SlCRK27	Solyc05g051270	280	8.34	30.02	23.32	0.136	Plasma membrane
SlCRK28	Solyc05g055930	282	5.73	31.95	36.52	-0.134	Plasma membrane
SlCRK29	Solyc06g083750	296	8.96	31.55	29.45	-0.114	Plasma membrane
SlCRK30	Solyc09g057960	261	8.67	29.53	35.26	-0.105	Plasma membrane
SlCRK31	Solyc09g065040	138	4.94	15.13	38.85	-0.059	Plasma membrane
SlCRK32	Solyc09g090680	329	8.69	35.09	34.3	0.101	Plasma membrane
SlCRK33	Solyc11g011000	283	6.91	30.85	38.9	0.073	Plasma membrane
SlCRK34	Solyc11g011870	454	7.97	50.29	37.58	-0.182	Plasma membrane
SlCRK35	Solyc11g011880	642	8.36	71.19	41.48	-0.193	Plasma membrane
SlCRK36	Solyc12g005720	240	5.17	27.01	40.49	-0.31	Plasma membrane
SlCRK37	Solyc12g087920	583	6.18	66.11	43.53	-0.161	Plasma membrane
